# Big data, privacy and COVID-19 – learning from humanitarian expertise in data protection

**DOI:** 10.1186/s41018-020-00072-6

**Published:** 2020-05-18

**Authors:** Andrej Zwitter, Oskar J. Gstrein

**Affiliations:** Data Research Centre, Campus Fryslân, University of Groningen, Leeuwarden, the Netherlands

## Abstract

The COVID-19 pandemic leads governments around the world to resort to tracking technology and other data-driven tools in order to monitor and curb the spread of SARS-CoV-2. Such large-scale incursion into privacy and data protection is unthinkable during times of normalcy. However, in times of a pandemic the use of location data provided by telecom operators and/or technology companies becomes a viable option. Importantly, legal regulations hardly protect people’s privacy against governmental and corporate misuse. Established privacy regimes are focused on individual consent, and most human rights treaties know derogations from privacy and data protection norms for states of emergency. This leaves little safeguards nor remedies to guarantee individual and collective autonomy. However, the challenge of responsible data use during a crisis is not novel. The humanitarian sector has more than a decade of experience to offer. International organisations and humanitarian actors have developed detailed guidelines on how to use data responsibly under extreme circumstances. This article briefly addresses the legal gap of data protection and privacy during this global crisis. Then it outlines the state of the art in humanitarian practice and academia on data protection and data responsibility during crisis.

## Introduction

On 11 March 2020 the World Health Organization declared that the spread of COVID-19 has resulted in a global pandemic (WHO Director-General’s Opening Remarks at the Media Briefing on COVID-19 - 11 March 2020 n.d). Since the virus gained international attention after its rapid spread in Hubei province in the People’s Republic of China (PRC) in December 2019, it subsequently appeared in other Asian countries such as South Korea and Japan. While some might argue that it was naïve of the ‘Western World’ to consider this crisis as a predominantly Asian problem for too long, the shutdown of large parts of society in practically all European countries and increasingly the rest of the world has made clear that this is a global crisis that affects all of us for much longer than expected. In this situation strong and decisive measures to save the lives and livelihoods of people across all parts of the world are needed.

However, more than ever before we are prepared to handle such crisis. Amongst others, Big data, artificial intelligence and blockchain technology can concretely help to deal with this emergency (Qadir et al. 2016). For example, location data from mobile phone companies can help in determining and understanding movement patterns of individuals and groups to potentially give insight into how the virus spreads and whether instructions are complied with (Ali et al. 2016). Governments and private corporations are developing apps that allow users to share their whereabouts and social contacts on a voluntary basis (Google Is Now Publishing Coronavirus Mobility Reports, Feeding off Users n.d.; Baharudin and Wong 2020); Experts Warn of Privacy Risk as US Uses GPS to Fight Coronavirus Spread 2020). Blockchain technology might be able to help keep a decentralised and cryptographically secure ledger of stocks and medication to create smart supply chain management (Zwitter and Boisse-Despiaux 2018). VR can help teachers explore new avenues of digital classrooms with new ways of interaction (Checa and Bustillo 2020). Nevertheless, as promising as the use of these emerging technologies might be, it is important to note that their use comes with a digital footprint that invariably has consequences for data protection and privacy – on a global scale (Zwitter 2015). Furthermore, political and corporate players might use the current situation to justify more intrusive data use for the future and for times after the pandemic is over.

This article will first discuss the potential and current use cases of location data for public order and specifically for getting the spread of COVID-19 under control. It will then outline concerns regarding ongoing practices. We will subsequently argue that these concerns are not mitigated by applicable data protection regimes or human rights norms due to their focus on the individual and respective derogation norms. In conclusion, we propose that guiding principles and standards for data practices in the humanitarian field are applicable during this crisis, and they should be considered as minimum standards for all states and corporations considering the use of data-driven monitoring tools to tackle the COVID-19 crisis.

## Location data, public order and control

When fighting a large-scale crisis such as a pandemic it is important for governments to understand why a threat is emerging, how the threat scenario develops, and whether the general population complies with measures for containment. Governments and research institutions need data to develop insights on these aspects, with location data being particularly attractive as work in the humanitarian sector has shown for many years by now.

When it comes to the use of location data sourced through mobile communication infrastructure and location services specifically, many commentators have been surprised by the fact that the government of the People’s Republic of China (PRC) co-developed a mobile phone application informing users whether they have been in close contact with someone infected by COVID-19 (China Launches Coronavirus ‘close Contact’ App 2020). The insights presented by this app are based on the analysis of location data collected through phone networks, WiFi connections, satellite-based radio navigation system (e.g. GPS, GLONASS**,** Galileo) and other surveillance assemblages producing data that reveal the location of individuals and crowds. Furthermore, apps with maps to track the disease also became popular very quickly in Hong Kong (Latest Situation of Novel Coronavirus Infection in Hong Kong n.d.), and South Korea (Coronavirus Mobile Apps Are Surging in popularity in South Korea - CNN n.d.). In the PRC, this approach seems to have evolved into the ‘Alipay Health Code’, a system that classifies residents based on an opaque methodology (Mozur et al. 2020). Once a survey has been filled out by a user, this data gets combined with other sources such as location data. Once the data has been analysed, a QR code is generated which has one of three colours; green enables its bearer to unrestricted movement, the ‘owner’ of a yellow code may be asked to stay home for 7 days, and a red QR code results in two-weeks of quarantine.

In the meanwhile, the US government is in active talks with several large technology corporations such as Google and Facebook to explore venues how location data could be used to combat the pandemic, including tracking whether people are keeping one another at safe distances to counter the spread of the virus (Romm et al. n.d.). Google has already used the pandemic to show some of the advantages of omnipresent location tracking (Google Is Now Publishing Coronavirus Mobility Reports, Feeding off Users’ Location History n.d.). Finally, surveillance corporations such as Athena Security and the infamous spyware firm NSO advertise specialized surveillance cameras and dedicated data analysis services using location data to track the spread of the disease based on the movement of individuals and groups (Cox 2020; Israel Spyware Firm NSO Wants to Track and Stop Coronavirus – Bloomberg n.d.).

## Human rights and data protection

Right now, the temptation is very strong to do “whatever is necessary” (Sevastopulo et al. 2020). Undoubtedly, in times of crisis there is an increased need for governments to monitor and control the public, which might make it necessary to limit individual freedom. Such decisionism characterizes many emergencies. Constitutions and human rights, however, are designed with such crises in mind. Furthermore, the International Covenant on Civil and Political Rights (ICCPR) and on the European level the European Convention on Human Rights (ECHR), are prepared to deal with such situations. Considering such developments from a formal perspective, it is useful to take a look at the legal and institutional framework of the Council of Europe (CoE). This international organization administers and controls one of the most important international human rights treaties guaranteeing individual freedoms, the European Convention on Human Rights (ECHR). The CoE has established procedures and case-law for times of crisis like the current one (Mokhtar 2004).

The guide on Article 15 ECHR for derogations in times of emergency has been updated recently on 31 December 2019 (Council of Europe/European Court of Human Rights 2019). States may derogate in situations of:

- war or other public emergency threatening the life of the nation,
- taking measures which are strictly required by the exigencies of the situation,
- and provided that measures are not inconsistent with other obligations under international law.

Furthermore, Article 4 of the ICCPR is similarly worded and beyond that requires state parties to report to all other parties via the UN Secretariat. Certain rights such as the right to life (except in respect of deaths resulting from lawful acts of war), the prohibition of torture and other forms of ill-treatment, the prohibition of slavery or servitude, and the rule of no punishment without law are non-derogable. However, many other rights are subject to derogation, including particularly the right to privacy, freedom of expression, the freedom of movement, as well as the freedom of assembly and association. Such derogations may only be of temporary nature (Zwitter 2012). Both of these legal frameworks allow states for some flexibility by enabling them to temporarily derogate from some rights.

Data protection and privacy are human rights that can be derogated from during crisis. They can be temporarily reduced when a public emergency calls for it. What makes this situation even more complicated is the use of data from and by corporate agencies. Only mentioning the issue of over-dominant corporate power in the form of surveillance capitalism briefly (Zuboff 2019), data ownership is in principle a matter of contract law and in many cases a question of terms of use that customers have to accept by default when intending to use a service. Particularly now, private corporations hold the key to using Big Data for tackling the corona crisis. Furthermore, typical data protection frameworks such as the EU General Data Protection Regulation (GDPR) are focused on individual rights and individual consent. Hence, they leave out many aspects of collective autonomy as outlined below. In summary, standard data protection regimes and human rights law provide little protection for privacy and responsible data use during times of emergency.

## Potential concerns

Over the last years much has been written about the balance between security and individual freedom, particularly on the false trade-off between privacy and security (Solove 2011). While a pandemic such as the spread of COVID-19 requires comprehensive measures, we must keep in mind that the use of location data and other (potentially) personally or demographically identifiable data on such scale results in the production of a ‘data exhaust’ that invariably has consequences. Just because it might be an emergency, does not mean that everything goes.

The arguably under-considered use of location data is surprising at this point when thinking about the unintentional revelation of the location and features of US military bases through the usage of the fitness app ‘Strava’ by members of the forces (Liptak 2018), or recent work of the New York Times based on the analysis of a comprehensive set of pseudonymized mobile phone records that allowed to identify several prominent and influential individuals upon closer scrutiny (Thompson and Warzel 2019). No executive powers enshrined in regulatory frameworks were necessary to acquire these datasets and carry out the analysis, which in itself shows that our societies lack appropriate governance frameworks for such practices. Not only effective oversight on the use of such data is missing, it is also open how individuals would be safeguarded against abuse, and which kind of remedies they could use to defend themselves. Considering this misuse of location data, the Federal Communications Commission in the US on 28 February 2020 proposed a fine of 200 million dollars for mobile phone network operators repackaging and reselling location data (FCC Proposes Over $200M in Fines for Wireless Location Data Violations 2020).

Furthermore, research over the past years has proven again and again that the combination of the production of unprecedented amounts of data and improving techniques to analyse large data sets are rendering most – if not all – state of the art practices to pseudonymize/anonymize datasets meaningless, at least as time moves on (Rocher et al. 2019). The United Nations Special Rapporteur on the right to privacy has rightfully highlighted the risks resulting from the combination of ‘closed’ datasets with ‘open’ ones (Cannataci 2017). In our work on Mobile devices as stigmatizing security sensors we have proposed the concept of ‘technological gentrification’ which describes our lives in environments that are permanently monitored and where those believing in the benefits of omnipresent data render the choices of others de-facto obsolete (Gstrein and van Eck 2018).

While a crisis like the coronavirus pandemic requires dedicated, quick and effective measures we must not forget that data is contextual. One and the same dataset can be sensitive in different contexts, and we need appropriate governance frameworks to make sure that this data is being generated, analysed, stored and shared in legitimate and responsible ways. In light of the COVID-19 pandemic location data might be very useful for epidemiological analysis. In the context of a political crisis, the same location data can threaten the rule of law, democracy and the enjoyment of human rights.

Luckily, some authorities across the world have already reacted to the potential threats resulting from the use of location data in order to tackle the current pandemic (Data Protection Law and the COVID-19 Outbreak n.d.). On 16 March 2020 the European Data Protection Board released a statement in which chair Andrea Jelinek underlines that “[…] even in these exceptional times, the data controller must ensure the protection of the personal data of the data subjects (Olbrechts 2020). Therefore, a number of considerations should be taken into account to guarantee the lawful processing of personal data. […].”

While these efforts are commendable, it would be preferable to have dedicated legal frameworks, created through democratic processes in parliaments, as well as transparent policies. Given the necessity to act quickly, one might at least expect governmental decrees or executive acts describing the means, objectives and undertaken practices in a detailed manner, rooted in proper legal basis and competences, including the establishment of oversight mechanisms. Instead, the current picture suggests that ad-hoc practices have to be justified by independent data protection authorities which have to compromise their long-term supervisory objectives for short-term support of the greater good.

## Humanitarian guidelines for data responsibility

Because of the lack of legal guidance in many instances, it is important to resort to best practices established in different fields, particularly in the domain of humanitarian action. Over the past decades the humanitarian community has developed extensive expertise on how to deal with data during crisis responsibly. One core player in this field is the United Nations’ Office for the Coordination of Humanitarian Affairs (OCHA). Its Centre for Humanitarian Data worked - together with many experts - on detailed guidance notes that help to deal with data responsibly (Data Policy – The Centre for Humanitarian Data n.d.). This particularly concerns best practices in the cooperation between humanitarian, corporate and governmental stakeholders.

The International Committee of the Red Cross and Red Crescent published a detailed handbook on data protection in humanitarian action (Kuner and Marelli 2017). This handbook covers everything from basic data protection principles, to questions of data sharing and data protection impact assessments (DPIA) in humanitarian contexts. Furthermore, Part II covers specific scenarios and data collection methods such as the use of mobile apps, biometrics and cloud services. These guidelines cover largely the same aspects as the OCHA guidelines. These concern particularly the fair data processing of vulnerable data subjects, data minimization as well as data retention and deletion.

Additionally, the Dutch Red Cross together with other Red Cross and Red Crescent societies initiated a group of expertise in the field of innovation and data science for humanitarian action, which issued the “510 Data Responsibility Policy” (Van Der Veen n.d.). This policy introduces key principles such as:

1. Data Protection,
2. Legality and Legitimacy,
3. Do No Harm,
4. Respect for the Rights of Data Subjects (including access, rectification and erasure),
5. Purpose Specification of Collected Data,
6. Minimisation (collection on the basis of necessity and proportionality), and
7. Data Quality as to accuracy, being up to date, valid, reliable and relevant.

One of the key findings is that data protection goes beyond the individual and includes vulnerable groups. This marks a shift from Personally Identifiable Data (PII) to Demographically Identifiable Data (DII) (Raymond 2017). Hence, data collection and utilization needs to follow the principle of proportionality and consider benefits and harms beyond individual interests. Furthermore, this thinking introduces a data lifecycle, which entails the stages of processing data from consideration of a potential data collection, over subsequent collection, to analysis and deletion of the data.

## Principles of data protection in humanitarian studies

At this stage it should be emphasized that the academic community has not been idle. A discussion surrounding data ethics has been held quite vigorous over past years. It ranges from questions surrounding the use of “public data” (e.g. social media data), to biases, and includes considerations on nudging (Boyd and Crawford 2012; Zwitter 2014; Zimmer 2010; Chandler 2015). Experts in the field of humanitarian action, innovation governance and data protection have published extensively on utilities and risks of the use of ‘big crisis data’. (Latif et al. 2019) A specific use-case has been the domain of crisis mapping, with Ushahidi and Humanitarian OpenStreetMap launching these developments early on (Ziemke 2012).

Of particular note in the past years has been the Signal Code of the Harvard Humanitarian Initiative (Greenwood et al. 2017). Its purpose is identification, definition and articulation of international Human Rights standards with regards to data and ICTs, as well as their translation into the humanitarian context. Like the principles of the 510 Data Responsibility, the Signal Code identifies a set of rights held by all data subjects including the protection of DII.

Some specific aspects of the Signal Code should be highlighted: The *right to information* refers to the right of all people to generate, access, acquire, transmit, and benefit from information during a crisis. The *right to protection* concerns protection from all harms that can arise from the misuse and unintended consequences of data and ICTs, given that crisis-affected populations are particularly vulnerable. *Privacy and security* as a right refers to internationally recognised legal, ethical and technical standards of data protection and privacy. The *right to data agency* relates to individual and collective agency with regards to collection, use and disclosure of PII and DII. Finally, *rectification and redress* of data is also a remedy that pertains to groups and individuals. A key element in all these points is not only the abstract existence and observance of these rights, but to enable effective application procedures for individuals and populations affected by crises. In other words, it obliges humanitarians to establish procedures to give effect to these rights and potential claims of affected people.

Further key areas of concern are (Karunakara 2013; Qadir et al. 2016; Gstrein and Zwitter n.d.; Ali et al. 2016):

1. the potential use of Big Data for unethical ends;
2. the potential to mislead through reliance on unrepresentative and biased data;
3. the various privacy and security challenges associated with data (including the danger data being tampered with),
4. and the erosion of humanitarian principles by the exploitative use of data through corporate agents.

Eventually, profound questions around the meaningfulness of concepts such as individual consent and the nature of effective pseudonymization and anonymization remain. Unfortunately, it goes beyond the scope of this short piece to explore these in detail, but considerations on ‘group privacy’ and informational self-determination in the digital age would be potential starting points for such in-depth discussion (Lu et al. 2012; Raymond 2017b; Taylor et al. 2017). It needs to be reiterated that the humanitarian field is working on this subject extensively and with a mindset that is focused on using data responsibly, instead of mere compliance with regulatory frameworks, which need to resort to abstract human rights provisions too quickly since these frameworks themselves are limited in scope and application. Hopefully, this gap can be filled quickly in order to be able to fully focus on the containment of the pandemic, instead of additionally creating worries around the responsible use of data.

## Conclusion

The use of location data to control the coronavirus pandemic can be fruitful and might improve the ability of governments and research institutions to combat the threat more quickly. It is important to note that location data is not the only useful data that can be used to curb the current crisis. Genetic data can be relevant for AI enhanced searches for vaccines and monitoring online communication on social media might be helpful to keep an eye on peace and security (Taulli n.d.). However, the use of such large amounts of data comes at a price for individual freedom and collective autonomy. The risks of the use of such data should ideally be mitigated through dedicated legal frameworks which describe the purpose and objectives of data use, its collection, analysis, storage and sharing, as well as the erasure of ‘raw’ data once insights have been extracted. In the absence of such clear and democratically legitimized norms, one can only resort to fundamental rights provisions such as Article 8 paragraph 2 of the ECHR that reminds us that any infringement of rights such as privacy need to be in accordance with law, necessary in a democratic society, pursuing a legitimate objective and proportionate in their application.

However as shown above, legal frameworks including human rights standards are currently not capable of effectively ensuring data protection, since they focus too much on the individual as the point of departure. Hence, we submit that currently applicable guidelines and standards for responsible data use in the humanitarian sector should also be fully applicable to corporate, academic and state efforts which are currently enacted to curb the COVID-19 crisis globally. Instead of ‘re-calibrating’ the expectations of individuals on their own privacy and collective autonomy, the requirements for the use of data should be broader and more comprehensive. Applicable principles and standards as developed by OCHA, the 510 project of the Dutch Red Cross, or by academic initiatives such as the Signal Code are valid minimum standards during a humanitarian crisis. Hence, they are also applicable minimum standards during the current pandemic.

Core findings that can be extracted from these guidelines and standards for the practical implementation into data driven responses to COVIC-19 are:

- **data sensitivity is highly contextual**; one and the same data can be sensitive in different contexts. Location data during the current pandemic might be very useful for epidemiological analysis. However, if (ab-)used to re-calibrate political power relations, data can be open for misuse. Hence, any party supplying data and data analysis needs to check whether data and insights can be misused in the context they are presented.
- **privacy and data protection are important values;** they do not disappear during a crisis. Nevertheless, they have to be weighed against respective benefits and risks.
- **data-breaches are inevitable**; with time (t) approaching infinity, the chance of any system being hacked or becoming insecure approaches 100%. Hence, it is not a question of whether, but when. Therefore, organisations have to prepare sound data retention and deletion policies.
- **data ethics is an obligation to provide high quality analysis;** using machine learning and big data might be appealing for the moment, but the quality of source data might be low, and results might be unreliable, or even harmful. Biases in incomplete datasets, algorithms and human users are abundant and widely discussed. We must not forget that in times of crisis, the risk of bias is more pronounced, and more problematic due to the vulnerability of data subjects and groups. Therefore, working to the highest standards of data processing and analysis is an ethical obligation.

The adherence to these principles is particularly relevant in times of crisis such as now, where they mark the difference between societies that focus on control and repression on the one hand, and those who believe in freedom and autonomy on the other. Eventually, we will need to think of including data policies into legal frameworks for state of emergency regulations, and coordinate with corporate stakeholders as well as private organisations on how to best deal with such crises. Data-driven practices have to be used in a responsible manner. Furthermore, it will be important to observe whether data practices and surveillance assemblages introduced under current circumstances will be rolled back to status quo ante when returning to normalcy. If not, our rights will become hollowed out, just waiting for the next crisis to eventually become irrelevant.
